# *Ophiorrhiza xishuiensis* (Rubiaceae), a new species endemic to Guizhou, Sichuan, and Chongqing, China

**DOI:** 10.3897/phytokeys.275.189435

**Published:** 2026-05-29

**Authors:** Xuan-Ze He, Zheng-Xian Dai, Jian Xu, Jia-Wen Yang, Sheng-Hu Tang

**Affiliations:** 1 Gesneriad Conservation Center of China (Guizhou), National Forestry and Grassland Administration Key Laboratory for Biodiversity Conservation in Karst Terrain of Southwestern China, Guizhou Botanical Garden, Guiyang 550000, China Gesneriad Conservation Center of China (Guizhou), National Forestry and Grassland Administration Key Laboratory for Biodiversity Conservation in Karst Terrain of Southwestern China, Guizhou Botanical Garden Guiyang China; 2 Guizhou Xishui National Nature Reserve Management Bureau, Xishui 564600, China Guizhou Xishui National Nature Reserve Management Bureau Xishui China

**Keywords:** Danxia landform, flora of China, new taxon, *
Ophiorrhiza
*

## Abstract

A new species in Rubiaceae, *Ophiorrhiza xishuiensis*, is described from China. The new species is endemic to the Danxia landform at the juncture of Guizhou Province, Sichuan Province, and Chongqing City. It is a herb with a 1–7 cm long rooting stem, 3–11 nodes, and most leaves arranged in markedly unequal pairs. It has a somewhat inequilateral leaf blade base, a congested-cymose inflorescence, and well-developed bracteoles. We investigated two populations in Guizhou, China. The new taxon has been collected or photographed by others in Sichuan and Chongqing, China, and misidentified as *O. japonica*, *O. umbricola*, *O. cantonensis*, or *O. chinensis*. An identification key to *O. xishuiensis* and morphologically similar species is provided.

## Introduction

The genus *Ophiorrhiza* L. is a part of the tribe Ophiorrhizeae, the subfamily Rubioideae, and the family Rubiaceae (Bremer and Manen 2000; Razafimandimbison and Rydin 2024). The species of *Ophiorrhiza* mainly occur in tropical and subtropical Asia and extend to Australia, New Guinea, and the Pacific islands (Darwin 1976; Lo 1999; Chen and Taylor 2011; Alfeche et al. 2020; Hareesh et al. 2020; Zhou et al. 2020). In China, comprehensive research on the genus *Ophiorrhiza* was done by Lo (1999) and Chen and Taylor (2011). Lo (1999) identified 72 species of *Ophiorrhiza* in China, and Chen and Taylor (2011) identified 70 species (49 endemic). In 2019, *Ophiorrhiza* was expanded to incorporate previously recognized genera (*Keenania* Hook.f. and *Spiradiclis* Blume) based on molecular evidence (Razafimandimbison and Rydin 2019). As of February 2026, the genus *Ophiorrhiza* includes 384 accepted species (POWO 2026), 140 of which native to China (Liu 2025).

In their key for Chinese species of *Ophiorrhiza*, Chen and Taylor (2011) attributed special importance to the following characters: length of calyx lobes, length of corolla tube, stipule and bracteole characters. Therefore, characters related to the calyx lobes, the corolla tube, the stipules and the bracteoles are key characteristics for identifying species of the genus *Ophiorrhiza* in China.

In June 2024, during a field survey in Guizhou Xishui National Nature Reserve, Guizhou Province, China, Xuan-Ze He discovered some small-sized plants of the genus *Ophiorrhiza*. He brought living plants back and cultivated them at Guizhou Botanical Garden. In March 2025, the cultivated plants bloomed, featuring well-developed bracteoles. Subsequently, we conducted another field survey to examine the wild plants. On 13 March 2025, we discovered flowering plants in Xishui County, Guizhou Province, that exhibited the same characteristics as the cultivated plants. On 14 March 2025, we discovered another population in Chishui City, Guizhou Province. Based on specimen and photographic records, we realized that the plants also occur in Xuyong County, Gulin County, Changning County, Weiyuan County, Xuzhou District, Jiangjin District, China. The plants are herbs with a 1–7 cm long rooting stem, 3–11 nodes, and most leaves arranged in markedly unequal pairs. They have somewhat inequilateral leaf blade bases, a congested-cymose inflorescence, and well-developed bracteoles. They are most similar to *O. japonica* Blume, *O. guizhouensis* C.D.Yang & G.Q.Gou, and *O. chinensis* H.S.Lo by the length of the stipules, calyx lobes and corolla tube, the shape of the corolla, with similar distribution areas and occurring at similar altitudes. However, after thorough comparison, we concluded that they represent a new species.

## Materials and methods

A population (including approximately 300 mature individuals) in Xishui County and another one (including approximately 15 mature individuals with long-styled flowers) in Chishui City have been investigated. Approximately 30 living plants from Xishui were cultivated in Guizhou Botanical Garden. Important morphological characteristics (e.g., the length of stem, peduncle, bracteoles and calyx lobes, the shape of leaf blade and flower characters) of approximately 100 mature individuals were observed. Approximately 20 short-styled flowers (all from the Xishui population) and 25 long-styled flowers (20 from the Xishui population, and 5 from the Chishui population) were observed and measured. A microscope (Olympus SZ61, Tokyo, Japan) was used for micro-observation. The species was described based on the two populations we investigated and the images of paratypes collected by others, following the terminology used by Chen and Taylor (2011).

Relevant literature was consulted, including Lo (1990, 1999), Chen and Taylor (2011), Hareesh and Sabu (2018), Tu et al. (2018), Yang et al. (2018), Duan et al. (2019), Hu et al. (2021), Liu et al. (2023, 2024), Shang et al. (2024), Zhan et al. (2024), and Xue et al. (2025). All species of *Ophiorrhiza* recorded in China have been examined. This was done using images of specimens or live plants, as well as the descriptions provided by Lo (1990, 1999) and Chen and Taylor (2011). The new taxon has been carefully compared with 27 similar species (Suppl. material 1: table SS1). Most images of specimens or live plants of these 27 similar species were sourced from virtual herbaria and databases (Suppl. material 1: table SS1), including iPlant (http://www.iplant.cn/), CVH (https://www.cvh.ac.cn/), CUBG (https://image.cubg.cn/), E (https://data.rbge.org.uk/search/herbarium/), K (http://apps.kew.org/herbcat/navigator.do), JSTOR (https://plants.jstor.org/), A (https://www.huh.harvard.edu/), BM (https://data.nhm.ac.uk/), and eFloraofIndia (https://efloraofindia.com/).

Twelve species of *Ophiorrhiza* have been grown at the Guizhou Botanical Garden, including *O. japonica* Blume, *O. guizhouensis* C.D.Yang & G.Q.Gou, *O. chinensis* H.S.Lo, *O. rufopunctata* H.S.Lo, and *O. cantonensis* Hance.

## Taxonomic treatment

### Ophiorrhiza xishuiensis

Taxon classification

Plantae

GentianalesRubiaceae

Sheng H.Tang, Jia W.Yang & X.Z.He sp. nov.

CB6434C8-C401-58E6-BF8A-AF4CC2894FCC

urn:lsid:ipni.org:names:77380759-1

Figs 1, 2, 3

#### Diagnosis.

The new species is most similar to *O. japonica*, *O. guizhouensis*, and *O. chinensis* by the length of the stipules, calyx lobes and corolla tube, the shape of the corolla, and the distribution areas and altitudes. It differs from *O. japonica* and *O. guizhouensis* by the 1–7 cm long rooting stems (vs. 10–30 cm, or longer), the fact that most leaves are arranged in markedly unequal (vs. subequal) pairs, the style reaching above (vs. below) the middle of the tube in the short-styled flowers, and the glabrous (vs. pubescent) style in the long-styled flowers. It is distinct from *O. chinensis* by the congested-cymose (vs. paniculiform to cymose) inflorescences, the well-developed and persistent (vs. absent or reduced and caducous) bracteoles, and the glabrous (vs. pubescent) style in the long-styled flowers.

**Figure 1.**
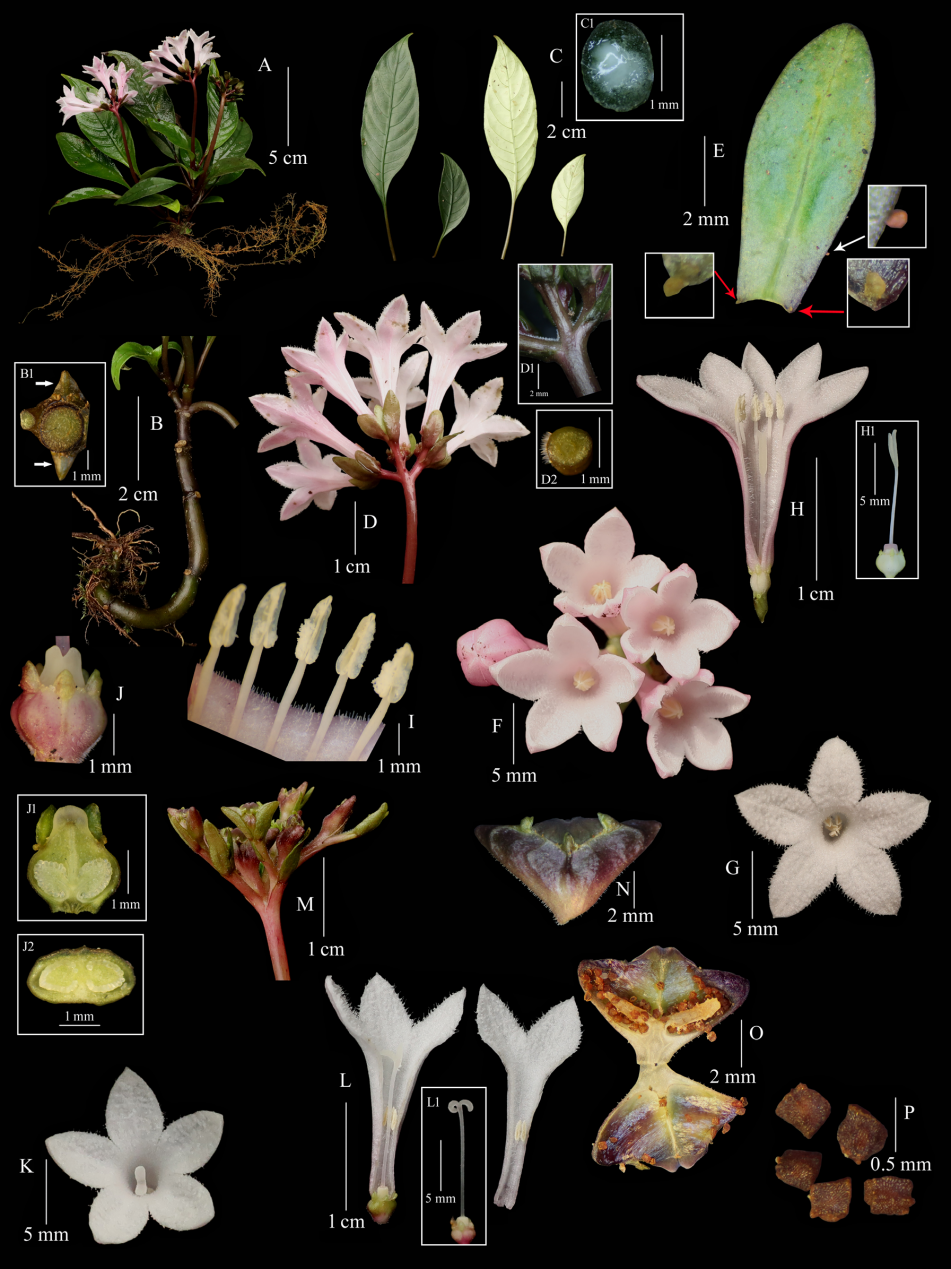
*Ophiorrhiza xishuiensis* sp. nov. **A**. Flowering plant; **B**. Stem and stipules indicated by white arrows (B1); **C**. Leaves in markedly unequal pairs and a very small amount of white sap from petiole (**C1**); **D**. Inflorescence (with part of the peduncle removed), inflorescence axes (**D1**), and axe cross-section (**D2**), showing the indumentum; **E**. Bracteoles and colleters (inset, indicated by arrows); **F, G**. Short-styled flowers in top view; **H**. Opened short-styled flower and pistil (**H1**); **I**. Stamens on the corolla tube in short-styled flower; **J**. Ovary, calyx, disk, base of style, ovary longitudinal section (**J1**), and ovary cross-section (**J2**); **K**. Long-styled flower in top view; **L**. Opened long-styled flower and pistil (**L1**); **M**. Infructescence (with part of the peduncle removed); **N**. Capsule; **O**. Opened capsule; **P**. Seeds (Photographed by Sheng-Hu Tang and Jia-Wen Yang).

**Figure 2.**
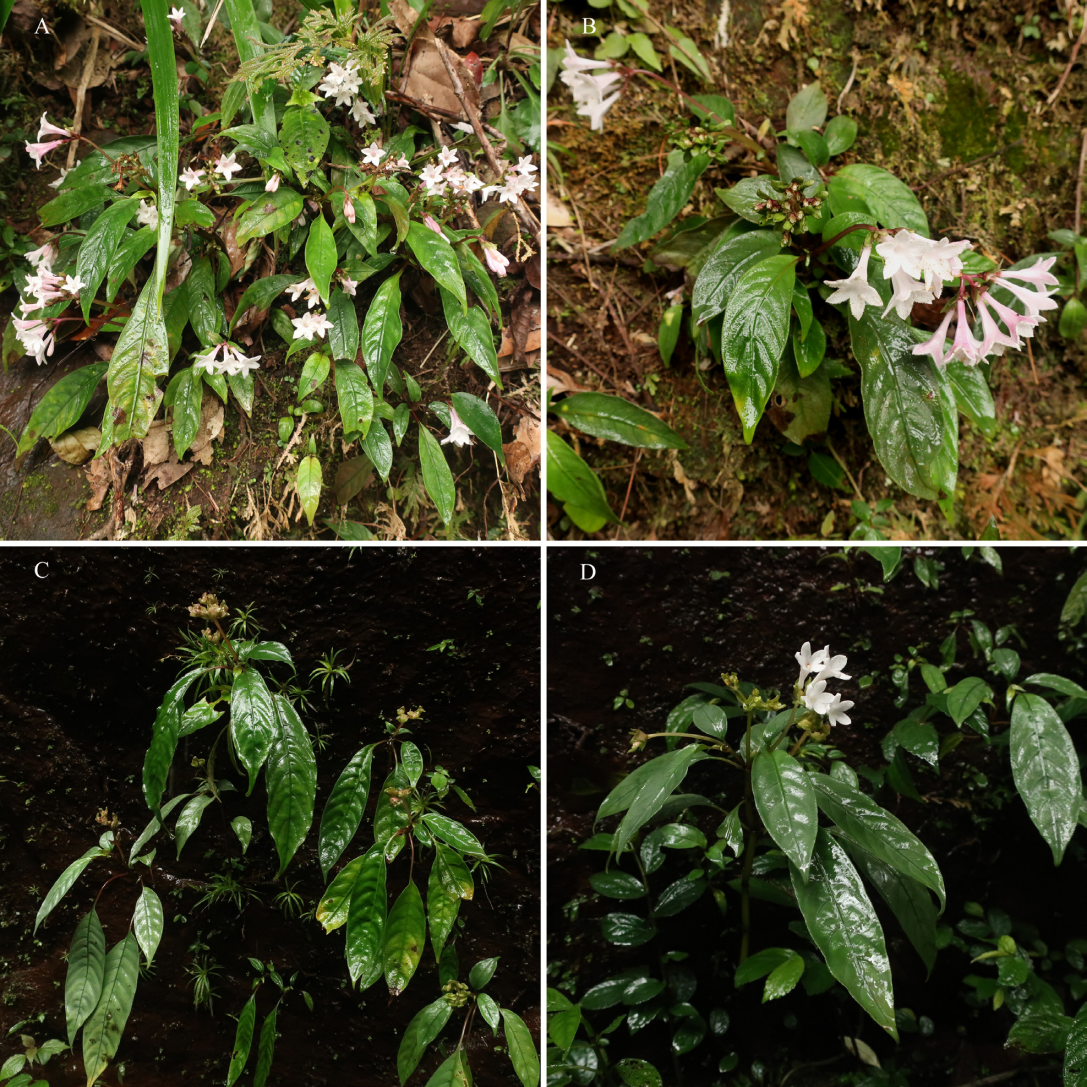
Habitat of *Ophiorrhiza xishuiensis* sp. nov. **A, B**. Population of Sanchahe Town, Xishui County, China; **C, D**. Population of Baoyuan Town, Chishui City, China (Photographed by Sheng-Hu Tang and Xuan-Ze He).

**Figure 3.**
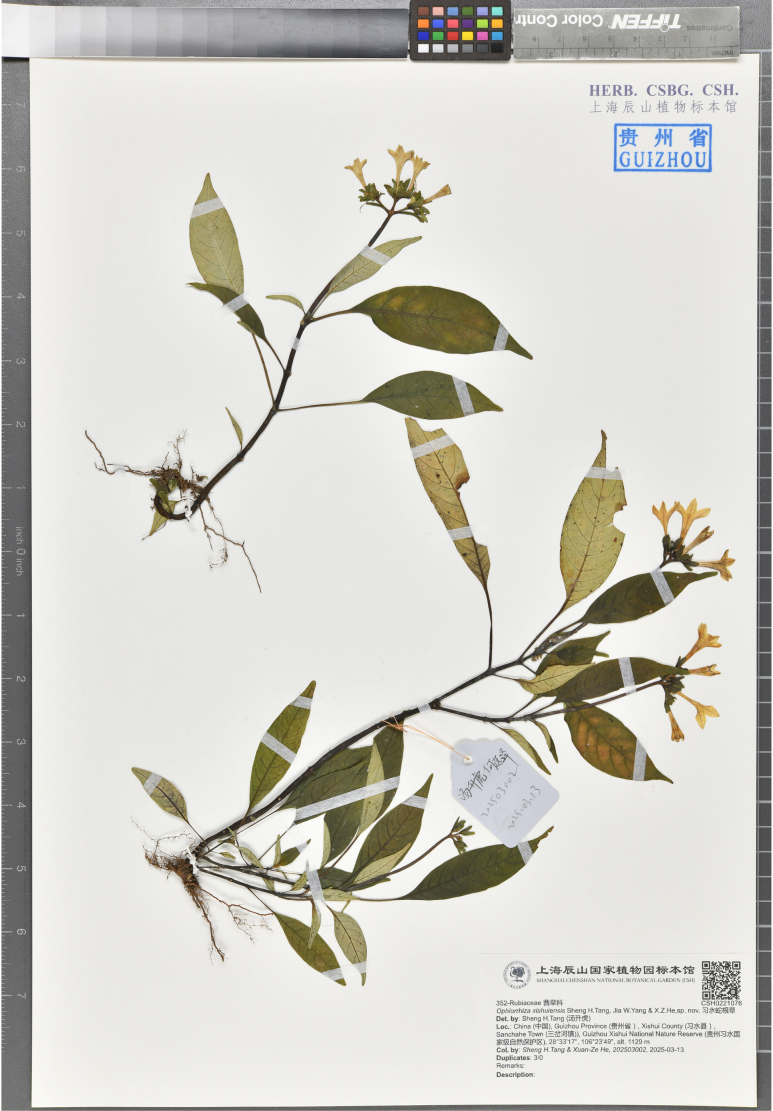
Holotype of *Ophiorrhiza xishuiensis* sp. nov. stored in CSH (*Sheng H.Tang & Xuan-Ze He 202503002*, CSH0221076).

#### Type.

**China** • Guizhou Province: Xishui County, Sanchahe Town, Guizhou Xishui National Nature Reserve, 28°33'17"N, 106°23'49"E, 1129 m, 13 March 2025, *Sheng H.Tang & Xuan-Ze He 202503002* (holotype: CSH! [Barcode: CSH0221076]; isotypes: CSH!, the Guizhou Botanical Garden!).

#### Description.

Perennial herbs, erect, 10–20 cm tall; stems glabrous, terete, 2.5–4.5 mm in diameter, 3–11 nodes, most internodes ascending and most nodes not rooting, rooting stem 1–7 cm long, creeping, a very small amount of white sap present when petiole is cut. Leaves mostly in markedly unequal pairs; petiole 0.5–4.5 cm long, glabrous, terete, 1–2 mm in diameter; blade drying papery, grayish green adaxially, pale green abaxially, oblanceolate, elliptic, lanceolate-elliptic, or oblong, larger ones 6–17 × 1.8–5 cm, smaller ones 1.5–5 × 0.5–1.5 cm, both surfaces glabrous or sometimes hirtellous adaxially, base cuneate, somewhat inequilateral, rarely subequilateral, margins entire, apex acute; secondary veins 6–9 pairs; stipules triangular, 0.8–1.2 mm long, caducous, with 5–6 globose colleters at the inner base. Inflorescences terminal, congested-cymose, 6–36 flowers per cyme, drooping at the early stage, then erect; peduncle 5–8 cm long, rarely shorter, 1.3–2.5 mm in diameter, glabrous; axes 5–10 mm long, congested-cymose, densely puberulent on the inside; bracts usually absent and sometimes well-developed, a little larger than bracteoles, bracteoles persistent, ovate, broadly ovate, or oblong, 4.7–12 × 2.2–6.5 mm, glabrous, margins entire with sparse colleters sometimes, apex obtuse, sometimes acute, midrib prominent on both sides, sparse colleters adaxially and abaxially sometimes, with 1 globose colleter at each side of inner base. Pedicels puberulent, 1–2 mm long or flowers subsessile. Flowers distylous. Hypanthium oblate, compressed, 1–1.4 × 2.4–2.8 mm, 5-ribbed, densely puberulent; calyx lobes 5, ovate, 0.5–1 × 0.5–0.7 mm, glabrous or sparsely puberulent outside, dorsally thickened, apex obtuse, with 1 globose colleter at each side of base outside and 1 globose colleter in each sinus. Corolla white or pale purplish red, drying pale yellow, tubular-funnelform, outside glabrous and longitudinally 5-ridged from apex to base; tube 9–16 mm long; lobes 5, triangular-ovate, 5–7 × 3.8–4.5 mm, adaxially scaly hairy and scaly glandular-hairy, apex acute, slightly incurved with a very short rostrum. Stamens 5, anthers linear, 2–2.3 mm long. Ovary 2-celled, densely puberulent to subglabrous. Long-styled flowers: corolla pubescent from below the middle up to the throat inside, without or with a white villous ring near middle of the tube; stamens inserted near the middle of the tube, filaments 0.3–0.4 mm long; style 9–12.6 mm long, glabrous, stigma positioned at the level of the throat or slightly exserted from the throat, lobes obovate or linear, 1.7–2.8 mm long. Short-styled flowers: corolla pubescent from below the middle up to the throat inside, without a white villous ring near the middle of the tube; stamens inserted in the throat, included or slightly exserted, filaments 1–1.2 mm long; style 4.5–7.5 mm long, reaching above the middle of the tube, glabrous, stigma positioned just below the throat, lobes linear, 3.2–3.4 mm long. Infructescence axes 8–16 mm long, densely puberulent on the inside. Capsules inverted triangle or submitriform, 4.7–5.7 × 7.8–10 mm, densely puberulent to subglabrous. Seeds numerous, rhomboid or nearly square, angled, 0.4–0.5 × 0.4–0.5 mm.

#### Phenology.

Flowering occurs from February to March, and fruiting occurs from April to July.

#### Etymology.

The new taxon was named after its type locality, Xishui County, Guizhou Province, China.

#### Vernacular name.

The Chinese name is “Xí Shuǐ Shé Gēn Cǎo” (习水蛇根草).

#### Distribution and habitat.

**Figure 4.**
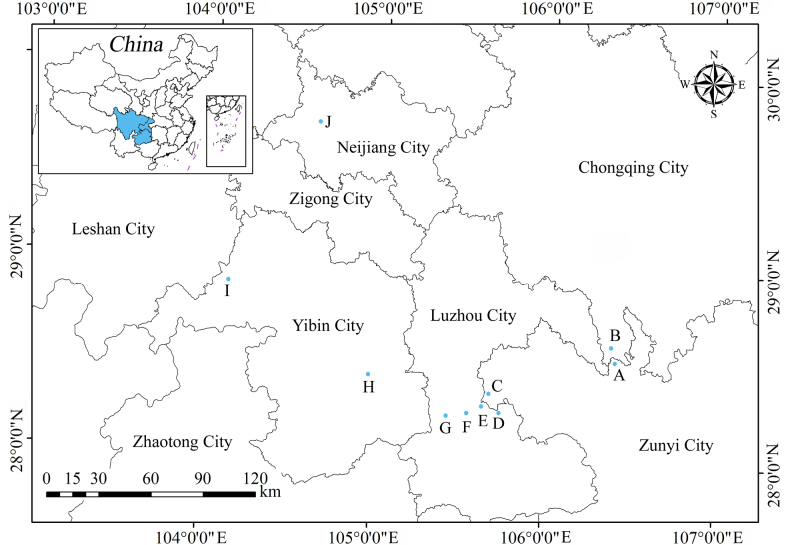
Locations of populations of *Ophiorrhiza xishuiensis* sp. nov. in Guizhou Province, Sichuan Province, and Chongqing City, China (indicated by the blue circles). The populations at point A and C have been investigated by us and point A is the type locality.

The species has been discovered in Guizhou Province (Xishui County, Chishui City), Sichuan Province (Xuyong County, Gulin County, Changning County, Weiyuan County, and Xuzhou District), and Chongqing City (Jiangjin District), China (Fig. 4). It grows in the Danxia landform, and thrives on moist, shady cliffs and sandy conglomerate surfaces. The Danxia landform is a red-bed landform characterized by steep cliffs.

#### Conservation status.

The species has been discovered in several counties and districts in Guizhou, Sichuan, and Chongqing, China. It is highly probable that additional populations exist in this region. Until further investigation is carried out, the species should be classified as “Data Deficient” (DD) following IUCN standards (IUCN 2024).

#### Additional specimens examined.

**China** • Sichuan Province: Gulin County, Huangjinglaolin Nature Reserve, 10 September 2013, *Yi-Hua Tong et al. 13091025* (BNU barcode BNU0018143 [photo!]); • Weiyuan County, Changtiankan, Guipiwan, *Weiyuan team 4029* (SM barcode SM718501570 & SM718501571 [photo!]); • Xuyong County, Shuiwei Town, Guangmu Village & Xixi Village, 1,000 m, 16 April 2014, *Lei Wu 4533* (BNU barcode BNU0018141 & BNU0018142 [photo!]); • Xuyong County, Shuiwei Town, during the journey from Guangmu Village (Group 2) to Suoluogou, 28°15'46"N, 105°31'0"E, 910 m, 13 June 2013, *Xin-Fen Gao, Zhang-Ming Zhu & Wen-Bin Ju HGX12115* (CDBI barcode CDBI0226195 [photo!]); • Xuyong County, Shuiwei Town, Guandou Village, 28°14'16"N, 105°38'11"E, 850 m, 7 June 2013, *Xin-Fen Gao, Yun-Dong Gao & Wen-Bin Ju HGX11695* (CDBI barcode CDBI0226617 [photo!]); • Xuyong County, Shuiwei Town, Guangmu Village, 28°09'37"N, 105°18'56"E, 1030 m, 20 February 2014, *Wen-Bin Ju HGX14071* (CDBI barcode CDBI0227705 & CDBI0227706 [photo!]); • Xuyong County, Longfeng Town, Siping Village, 28°09'02"N, 105°16'57"E, 960 m, 22 February 2014, *Wen-Bin Ju HGX14135* (CDBI barcode CDBI0226957 & CDBI0226958 [photo!]); • Changning County, Wanling Town (Wanling Commune), 600 m, 22 June 1977, *s.coll. 0479* (SM barcode SM718501565 [photo!]); • Xuzhou District, Longchi Town (Guangming Commune), Honghua Village, 500 m, 16 July 1977, *Yibin team 539* (SM barcode SM718501573 & SM718501574 [photo!]); • Guizhou Province: Chishui City, Baoyuan Town, Lianhua Village, 28°20'34"N, 105°39'46"E, 448 m, 14 March 2025, *Sheng H.Tang & Da-Zhu Tang 202503005* (the Guizhou Botanical Garden!).

#### Additional photographs examined.

China. Chongqing City: Jiangjin District, Simianshan Scenic Area, Xiang Liu (PPBC ID 21102971); Yong-Sheng Wei (PPBC ID 20813977, 20813979, 20813983, 20830320, 20830324, 20830330, 20830335, 20830339, 20830343, 20830353, 20848643, 20848645, 20848647, 20848649, 20848651, 20848655, 20848658).

#### Notes.

**Figure 5.**
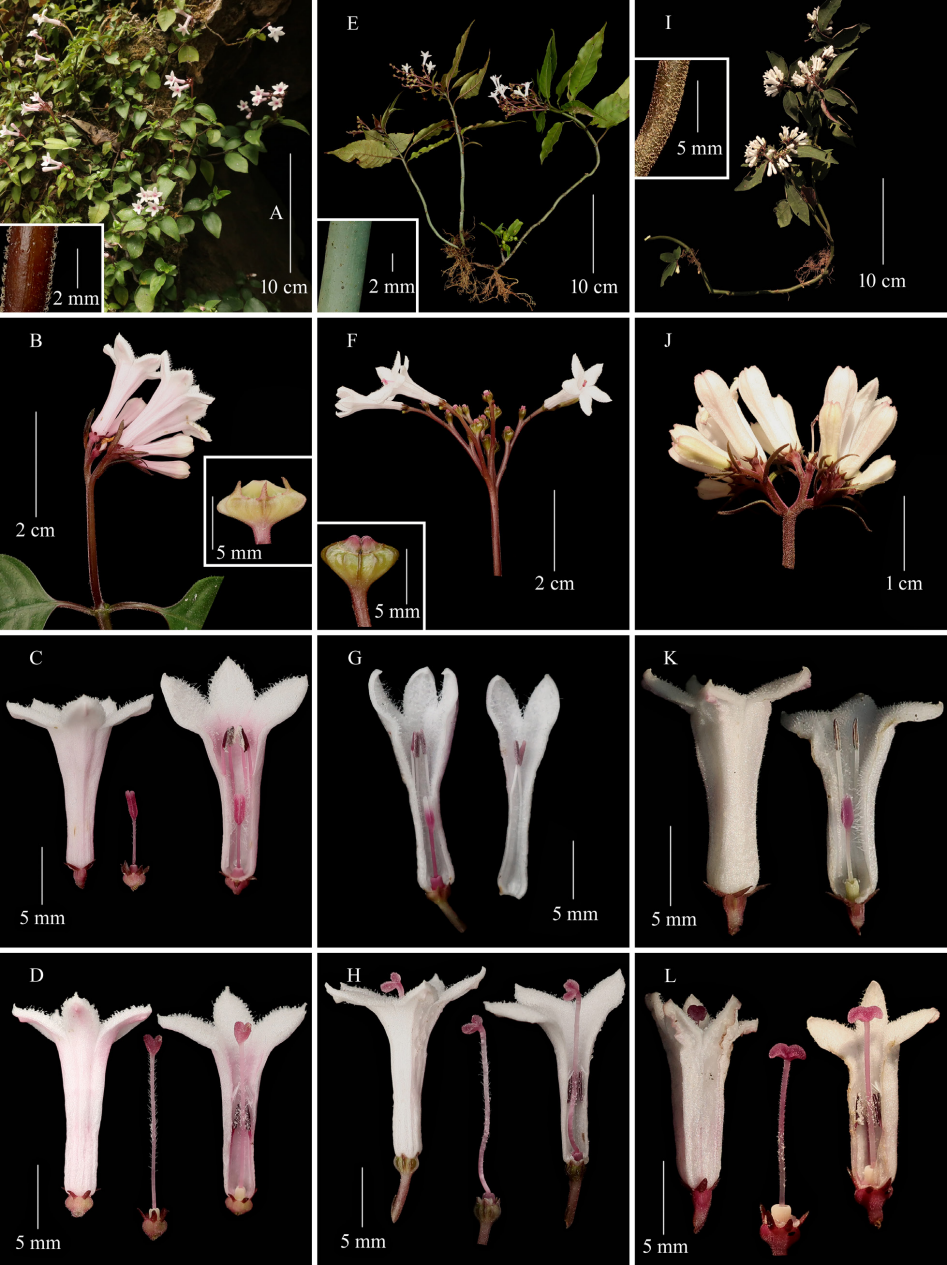
*Ophiorrhiza japonica* (**A–D**), *O. chinensis* (**E–H**) and *O. guizhouensis* (**I–L**). **A, E, I**. Flowering plants and a part of stem (inset); **B, F, J**. Inflorescence and young fruit (inset); **C, G, K**. Short-styled flowers; **D, H, L**. Long-styled flowers. (Photographed by Sheng-Hu Tang).

The new species is most similar to *Ophiorrhiza japonica* (Fig. 5), *O. guizhouensis* (Fig. 5), and *O. chinensis* (Fig. 5) by the length of stipules, calyx lobes and corolla tube, the shape of the corolla, and the distribution areas and altitudes. The new taxon is also similar to *O. umbricola* W.W.Sm., *O. grandibracteolata* F.C.How ex H.S.Lo and *O. liuyanii* L.Wu, Y.H.Tan & K.S.Nguyen by the congested-cymose inflorescences and well-developed bracteoles. It differs from *O. umbricola* by the 1–7 cm long rooting stems (vs. usually 15 cm, or longer), the somewhat inequilateral, rarely subequilateral leaf blade bases (vs. subequilateral), the 5–8 cm long (rarely shorter) peduncle (vs. 1.5–3 cm long). It can be distinguished from *O. grandibracteolata* by the glabrous (vs. densely villous with trichomes multicellular or sometimes also unicellular) stems, the 5–8 cm long (rarely shorter) and glabrous peduncle (vs. 0.6–1.7 cm long and densely multicellular villous), and the 9–16 mm long corolla tube (vs. 22–25 mm long). It is different from *O. liuyanii* by the fact that most leaves are arranged in markedly unequal (vs. equal) pairs, the 5–8 cm long (rarely shorter) peduncle (vs 1–2 cm long), the longitudinally 5-ridged corolla tube (vs. 5-winged), and the glabrous (vs. pubescent) style in the long-styled flowers. The new taxon is also similar to *O. cantonensis* Hance by the length of the corolla. It differs from *O. cantonensis* by the congested-cymose (vs. paniculiform to corymbose) inflorescences, the ovate, oblong, or broadly ovate (vs. linear) bracteoles, and the spreading (vs. reflexed) corolla lobes. A detailed morphological comparison is shown in Table 1. An identification key to *O. xishuiensis* and its morphologically similar species is provided.

**Table 1.** Detailed comparisons among *Ophiorrhiza xishuiensis*, *O. japonica*, *O. guizhouensis*, *O. chinensis*, *O. cantonensis*, *O. umbricola*, *O. grandibracteolata* and *O. liuyanii*.

**Characters**	***O. xishuiensis***	***O. japonica***	***O. guizhouensis***	***O. chinensis***	***O. cantonensis***	***O. umbricola***	***O. grandibracteolata***	***O. liuyanii***
Rooting stem	1–7 cm long	10–30 cm, or longer	10–30 cm, or longer	1–7 cm long, rarely longer	unknown	usually 15 cm, or longer	unknown	unknown
Stems	glabrous	glabrous or with 2 hirtellous or pilosulous lines	densely hirtellous	subglabrous to pilosulous	glabrous to densely puberulent or villosulous	glabrous or subglabrous	densely villous with trichomes multicellular or sometimes also unicellular	glabrous
Leaves	mostly arranged in markedly unequal pairs, base somewhat inequilateral, rarely subequilateral	arranged in subequal pairs, base subequilateral	arranged in subequal pairs, base subequilateral	arranged in subequal pairs, base subequilateral	arranged in subequal pairs, base subequilateral	mostly arranged in unequal pairs, base subequilateral	mostly arranged in markedly unequal pairs, base somewhat inequilateral	mostly arranged in equal pairs, base subequilateral
Bracteoles	persistent, ovate, broadly ovate, or oblong, 4.7–12 mm long	persistent, lanceolate-linear, spatulate, or linear, 1–6 mm long	persistent, lanceolate-linear, spatulate, or linear, 1–6 mm long	absent or reduced (caducous, linear, 1–1.5 mm long)	persistent, linear, 1–6 mm long	persistent, elliptic or oblong, 4–7 mm long	persistent, ovate, 10–15 mm long	persistent, ovate or broadly ovate, 9–22 mm long
Inflorescence	congested-cymose	congested-cymose to cymose	congested-cymose to cymose	paniculiform to cymose	paniculiform to corymbose	congested-cymose	congested-cymose	congested-cymose
Inflorescence axes	congested-cymose	congested-cymose or helicoid	congested-cymose or helicoid	helicoid	helicoid	congested-cymose	congested-cymose	congested-cymose
Peduncle	5–8 cm long, rarely shorter	0.5–6 cm long	1–2 cm long	1.5–3.5 cm long	1.5–7 cm long	1.5–3 cm long	0.6–1.7 cm long	1–2 cm long
Style in short-styled flowers	reaching above the middle of the tube	reaching below the middle of the tube	reaching below the middle of the tube	reaching below the middle of the tube	reaching below the middle of the tube	unknown	unknown	reaching below the middle of the tube
Style in long-styled flowers	glabrous	pubescent	pubescent	pubescent	unknown	unknown	unknown	pubescent

##### Key to *Ophiorrhiza xishuiensis* and its morphologically similar species

**Table:** 

1	Inflorescence paniculiform, corymbose or cymose, and inflorescence axes helicoid	**2**
–	Inflorescence congested-cymose, and inflorescence axes congested-cymose	**4**
2	Calyx lobes 0.7–1.2 mm long in flowers, and 1.5–2 mm long in fruits	***O. japonica***
–	Calyx lobes 0.4–0.5 mm long in flowers, and 0.6–0.8 mm long in fruits	**3**
3	Corolla lobes reflexed, bracteoles linear and persistent	***O. cantonensis***
–	Corolla lobes spreading, bracteoles absent or linear and caducous	***O. chinensis***
4	Bracteoles ovate, broadly ovate, elliptic or oblong	**5**
–	Bracteoles lanceolate-linear, spatulate or linear	**8**
5	Stems densely villous with trichomes multicellular or sometimes also unicellular	***O. grandibracteolata***
–	Stems glabrous or subglabrous	**6**
6	Peduncle 5–8 cm long, rarely shorter, leaf blade base somewhat inequilateral	***O. xishuiensis* sp. nov**.
–	Peduncle 1–3 cm long, leaf blade base subequilateral	**7**
7	Bracteoles ovate or broadly ovate, corolla tube outside longitudinally winged	***O. liuyanii***
–	Bracteoles elliptic or oblong, corolla tube outside longitudinally 5-ridged	***O. umbricola***
8	Stems densely hirtellous	***O. guizhouensis***
–	Stems glabrous or with 2 hirtellous or pilosulous lines	***O. japonica***

*Ophiorrhiza* typically prefers to grow near streams or in damp areas. Few people have paid attention to its sap. We examined the new taxon and *O. japonica* and found that the new taxon has a very small amount of white sap, while the latter has a very small amount of transparent, water-like sap, which becomes visible when cutting the petiole.

*Ophiorrhiza* often has colleters inside the stipules and calyx. However, the colleters inside or on the bracts and bracteoles have often been overlooked. We examined the new taxon and *O. japonica* and found that both have bracteoles with a single globose colleter (yellow in colour) on each side of the inner base. Sometimes, the new taxon has red sparse colleters on both the adaxial and abaxial surfaces of the bracteoles, as well as on the margin, while *O. japonica* has yellow sparse colleters. Further research on the presence/absence and position of colleters in the genus is needed to better understand the taxonomical value of this character.

## Supplementary Material

XML Treatment for Ophiorrhiza xishuiensis

## Additional information

### Artificial Intelligence (AI) use

The authors accept full responsibility for the content of the manuscript, including the disclosure of any use of AI.

No AI tools were used in the preparation of this manuscript.

### Author ORCIDs

X-Z. He https://orcid.org/0009-0002-6432-1834

Z-X. Dai https://orcid.org/0009-0003-7938-7243

J. Xu https://orcid.org/0000-0002-0714-0917

J-W. Yang https://orcid.org/0000-0003-3427-714X

S-H. Tang https://orcid.org/0000-0002-2168-8559

### Data availability

All of the data that support the findings of this study are available in the main text or Supplementary Information.

## Supplementary materials

Supplementary material 1 Supplementary table SS1 Data type pdf Explanation note Images of living plants or specimens (*Ophiorrhiza xishuiensis* and 27 similar species) examined. https://binary.pensoft.net/file/1662123This dataset is made available under the Open Database License (http://opendatacommons.org/licenses/odbl/1.0/). The Open Database License (ODbL) is a license agreement intended to allow users to freely share, modify, and use this Dataset while maintaining this same freedom for others, provided that the original source and author(s) are credited. Xuan-Ze He, Zheng-Xian Dai, Jian Xu, Jia-Wen Yang, Sheng-Hu Tang

Supplementary material 2 Supplementary figures S1–S4 Data type pdf Explanation note Plants of the new species cultivated in the Guizhou Botanical Garden. https://binary.pensoft.net/file/1662124This dataset is made available under the Open Database License (http://opendatacommons.org/licenses/odbl/1.0/). The Open Database License (ODbL) is a license agreement intended to allow users to freely share, modify, and use this Dataset while maintaining this same freedom for others, provided that the original source and author(s) are credited. Xuan-Ze He, Zheng-Xian Dai, Jian Xu, Jia-Wen Yang, Sheng-Hu Tang
