# Supplementary material for: Ophiorrhiza xishuiensis (Rubiaceae), a new species endemic to Guizhou, Sichuan, and Chongqing, China
Source: PhytoKeys. 2026 May 29;275:163–76. doi: 10.3897/phytokeys.275.189435 (PMC13241921; doi:10.3897/phytokeys.275.189435)
Supplement: Supplementary material 1 — Supplementary table SS1 [file phytokeys-275-163_article-189435__-s001.pdf]

Supplementary Material 1. Images of living plants or specimens (*Ophiorrhiza xishuiensis* and 27 similar species) examined			
Species	Collector and number or photographer	Barcode or ID	Available from
Ophiorrhiza xishuiensis	Yi-Hua Tong et al. 13091025	BNU0018143	https://www.cvh.ac.cn/spms/detail.php?id=efad68ba
Ophiorrhiza xishuiensis	Weiyuan team 4029	SM718501570	https://www.cvh.ac.cn/spms/detail.php?id=f71f10d0
Ophiorrhiza xishuiensis	Weiyuan team 4029	SM718501571	https://www.cvh.ac.cn/spms/detail.php?id=f71f116c
Ophiorrhiza xishuiensis	Lei Wu 4533	BNU0018141	https://www.cvh.ac.cn/spms/detail.php?id=efad677b
Ophiorrhiza xishuiensis	Lei Wu 4533	BNU0018142	https://www.cvh.ac.cn/spms/detail.php?id=efad6818
Ophiorrhiza xishuiensis	Xin-Fen Gao, Zhang-Ming Zhu & Wen-Bin Ju HGX12115	CDBI0226195	https://www.cvh.ac.cn/spms/detail.php?id=f7d53dec
Ophiorrhiza xishuiensis	Xin-Fen Gao, Yun-Dong Gao & Wen-Bin Ju HGX11695	CDBI0226617	https://www.cvh.ac.cn/spms/detail.php?id=45e79e50
Ophiorrhiza xishuiensis	Wen-Bin Ju HGX14071	CDBI0227705	https://www.cvh.ac.cn/spms/detail.php?id=f7dc5802
Ophiorrhiza xishuiensis	Wen-Bin Ju HGX14071	CDBI0227706	https://www.cvh.ac.cn/spms/detail.php?id=4645f65b
Ophiorrhiza xishuiensis	Wen-Bin Ju HGX14135	CDBI0226957	https://www.cvh.ac.cn/spms/detail.php?id=46485985
Ophiorrhiza xishuiensis	Wen-Bin Ju HGX14135	CDBI0226958	https://www.cvh.ac.cn/spms/detail.php?id=f7dc90d7
Ophiorrhiza xishuiensis	s.coll. 0479	SM718501565	https://www.cvh.ac.cn/spms/detail.php?id=f70cd9b0
Ophiorrhiza xishuiensis	Yibin team 539	SM718501573	https://www.cvh.ac.cn/spms/detail.php?id=f70b0e04
Ophiorrhiza xishuiensis	Yibin team 539	SM718501574	https://www.cvh.ac.cn/spms/detail.php?id=f70b0ea4
Ophiorrhiza xishuiensis	Xiang Liu	21102971	https://ppbc.iplant.cn/tu/21102971
Ophiorrhiza xishuiensis	Yong-Sheng Wei	20813977	https://ppbc.iplant.cn/20813977 (https://ppbc.iplant.cn/ Additional ID: 20813979, 20813983, 20830320, 20830324, 20830330, 20830335, 20830339, 20830343, 20830353, 20848643, 20848645, 20848647, 20848649, 20848651, 20848655, 20848658)

O. hayatana Ohwi	Fan-Ming Kong	3445931	https://ppbc.iplant.cn/tu/3445931
O. hayatana Ohwi	Fan-Ming Kong	3445934	https://ppbc.iplant.cn/tu/3445934
O. hayatana Ohwi	Fan-Ming Kong	3445937	https://ppbc.iplant.cn/tu/3445937
O. hayatana Ohwi	Ching-I Peng 13800	00808283	https://www.cvh.ac.cn/spms/detail.php?id=f119de6f
O. japonica Blume	Miyoshi Furuse 8323	01271663	https://www.cvh.ac.cn/spms/detail.php?id=ed89289f
O. succirubra King ex Hook.f.	J.D. Hooker 17	K000031162	https://plants.jstor.org/fsi/img/size2/alukaplant/k/phase_01/k0002/k000031162.jpg
O. succirubra King ex Hook.f.	Meng-Qi Han & Fang-Pu Liu DHL71014	02383935	https://www.cvh.ac.cn/spms/detail.php?id=eef7fc2c
O.nanlingensis L.Wu & Q.R.Liu	Lei Wu	Not available	Hu YH, Liu WJ, Song XF, Deng GX, Nakamura K, Wu L, Liu QR (2021) A discussion of the relationship between *Ophiorrhiza exigua* and *O. michelloides* (Rubiaceae) with the description of a new species. Nordic Journal of Botany 39 (6): e03138. https://doi.org/10.1111/njb.03138
O. loana Y.F.Deng & Y.Feng Huang	Gui-Li Feng 4-10-321	GXMI050466	https://www.cvh.ac.cn/spms/detail.php?id=09175c08
O. loana Y.F.Deng & Y.Feng Huang	Gui-Li Feng 4-10-321	GXMI050469	https://www.cvh.ac.cn/spms/detail.php?id=09175da6
O. carnosicaulis H.S.Lo	Drawn by Han-Ping Yu	Not available	https://www.iplant.cn/info/Ophiorrhiza%20carnosicaulis?t=z
O. cantonensis Hance	G. Theophilus Sampson s.n.	BM000901218	https://data.nhm.ac.uk/dataset/56e711e6-c847-4f99-915a-6894bb5c5dea/resource/05ff2255-c38a-40c9-b657-4ccb55ab2feb/record/4995928
O. cantonensis Hance	Ang Liu	4579433	https://ppbc.iplant.cn/tu/4579433
O. cantonensis Hance	Ang Liu	4579435	https://ppbc.iplant.cn/tu/4579435
O. cantonensis Hance	Ang Liu	4579445	https://ppbc.iplant.cn/tu/4579445
O. cantonensis Hance	Ang Liu	4579447	https://ppbc.iplant.cn/tu/4579447
O. densa H.S.Lo	K.M. Feng 12918	0005848	https://image.cubg.cn/upload/m_photos/midthumb/20210617/7T377K53493577m78913X578s447o330p.jpg
O. densa H.S.Lo	K.M. Feng 12918	1209988	https://image.cubg.cn/upload/m_photos/midthumb/20210617/2Y940j65371858q26149I891i127A546B.jpg
O. densa H.S.Lo	Xin-Xin Zhu	4892437	https://ppbc.iplant.cn/tu/4892437
O. densa H.S.Lo	Xin-Xin Zhu	3211566	https://ppbc.iplant.cn/tu/3211566
O. densa H.S.Lo	Xin-Xin Zhu	4892435	https://ppbc.iplant.cn/tu/4892435
O. alatiflora H.S.Lo	Lei Wu	Not available	Shang C, Xue J, Yang YJ, Liao XW, Liu QR, Wu L (2024) *Ophiorrhiza reflexa* (Rubiaceae), a new species from a karst region in Guangxi, China. PhytoKeys 238: 231–240.

			https://doi.org/10.3897/phytokeys.238.116767
O. laevifolia H.S.Lo	Meng Li	18649300	https://ppbc.iplant.cn/tu/18649300
O. laevifolia H.S.Lo	Zi Wang	16872393	https://ppbc.iplant.cn/tu/16872393
O. laevifolia H.S.Lo	Meng Li	18649363	https://ppbc.iplant.cn/tu/18649363
O. laevifolia H.S.Lo	Zi Wang	16872394	https://ppbc.iplant.cn/tu/16872394
O. laevifolia H.S.Lo	Meng Li	18649401	https://ppbc.iplant.cn/tu/18649401
O. laevifolia H.S.Lo	Meng Li	18649412	https://ppbc.iplant.cn/tu/18649412
O. subrubescens Drake	B. Balansa 2741	K000740651	https://species.data.kew.org/species/urn:lsid:ipni.org:names:758345-1#
O. subrubescens Drake	You-Pai Zeng	19062500	https://ppbc.iplant.cn/tu/19062500
O. subrubescens Drake	You-Pai Zeng	19062483	https://ppbc.iplant.cn/tu/19062483
O. subrubescens Drake	You-Pai Zeng	19062488	https://ppbc.iplant.cn/tu/19062488
O. oppositiflora Hook.f.	C.B. Clarke 21878B	K000031233	https://species.data.kew.org/species/urn:lsid:ipni.org:names:758547-1
O. oppositiflora Hook.f.	C.B. Clarke 38062A	K0005222694	https://species.data.kew.org/species/urn:lsid:ipni.org:names:758547-1
O. oppositiflora Hook.f.	Not available	Not available	https://efloraofindia.com/wp-content/uploads/2020/10/Ophiorhiza%20sp%20-2-.JPG
O. alata Craib	A.F.G. Kerr 9643	K000740569	https://species.data.kew.org/species/urn:lsid:ipni.org:names:758353-1
O. chingii H.S.Lo	R.C. Ching 22367	0005846	https://image.cubg.cn/upload/m_photos/midthumb/20210617/3b476k52130228n20434s418H745X454K.jpg
O. laoshanica H.S.Lo	Zhao-Qian Zhang 11030	1219643	https://image.cubg.cn/upload/m_photos/midthumb/20210617/0w435U81936318o57703C448N423N230p.jpg
O. filibracteolata H.S.Lo	Yue71 24	0005850	https://image.cubg.cn/upload/m_photos/midthumb/20210617/6m602N94839338a51958W325S882j744z.jpg
O. filibracteolata H.S.Lo	Li Li	22935510	https://ppbc.iplant.cn/tu/22935510
O. filibracteolata H.S.Lo	Li Li	22935506	https://ppbc.iplant.cn/tu/22935506
O. filibracteolata H.S.Lo	Li Li	22935471	https://ppbc.iplant.cn/tu/22935471
O. petrophila H.S.Lo	H.S. Lo 2016	0473910	https://image.cubg.cn/upload/m_photos/midthumb/20210617/2E869H67213197y48377y449U229y705h.jpg
O. petrophila H.S.Lo	Xin-Xin Zhu, Jun Wang, Bo Xiao & Quan-Long Zhao	1439808	https://www.cvh.ac.cn/spms/detail.php?id=9be27f77

	ZXX19018		
O. petrophila H.S.Lo	Xin-Xin Zhu	4894328	https://ppbc.iplant.cn/tu/4894328
O. petrophila H.S.Lo	Xin-Xin Zhu	4894337	https://ppbc.iplant.cn/tu/4894337
O. paralatiflora L.Wu & Q.R.Liu	Lei Wu, Long-Fei Fu & Xiong Li	Not available	Zhan M, Liao XW, Song F, Xue L, Liu QR, Wu L (2024) *Ophiorrhiza paralatiflora* (Rubiaceae), a new species from limestone areas in Guangxi, China. Nordic Journal of Botany 2024 (7): e04391. https://doi.org/10.1111/njb.04391
O. dianguiensis L.Wu & Q.R.Liu	Lei Wu	Not available	Xue J, Liao XW, Nguyen KS, Song F, Wu L (2025) *Ophiorrhiza dianguiensis* (Rubiaceae), a new species from southwestern China. Phytotaxa 720 (1): 075–080. https://doi.org/10.11646/phytotaxa.720.1.7
O. liuyanii L.Wu, Y.H.Tan & K.S.Nguyen	Lei Wu, Xiao-Wen Liao & Khang Sinh Nguyen	Not available	Liu CY, Liao XW, Ye LC, Tan YH, Nguyen KS, Thien TD, Wu L (2024) *Ophiorrhiza liuyanii* (Rubiaceae), a new species from south-western China and northern Vietnam. PhytoKeys 248: 199–206. https://doi.org/10.3897/phytokeys.248.135078
O. reflexa L.Wu & Q.R.Liu	Lei Wu	Not available	Shang C, Xue J, Yang YJ, Liao XW, Liu QR, Wu L (2024) *Ophiorrhiza reflexa* (Rubiaceae), a new species from a karst region in Guangxi, China. PhytoKeys 238: 231–240. https://doi.org/10.3897/phytokeys.238.116767
O. umbricola W.W.Sm.	F. Kingdon-Ward 1944	E00326820	https://data.rbge.org.uk/search/herbarium/?specimen_num=378963&filename=E00326820.zip&cfg=zoom.cfg
O. umbricola W.W.Sm.	G. Forrest 17656	E00123925	https://data.rbge.org.uk/search/herbarium/?specimen_num=120607&filename=E00123925.zip&cfg=zoom.cfg
O. umbricola W.W.Sm.	G. Forrest 17656	E00143146	https://data.rbge.org.uk/search/herbarium/?specimen_num=120608&filename=E00143146.zip&cfg=zoom.cfg
O. chinensis H.S.Lo	Ze-Tang Li 1689A	0005844	https://image.cubg.cn/upload/m_photos/midthumb/20210617/2G825T63176082Z11949e373m112t947g.jpg
O. chinensis H.S.Lo	Jia-Hua Cai	13361977	https://ppbc.iplant.cn/tu/13361977
O. chinensis H.S.Lo	Jia-Hua Cai	13361999	https://ppbc.iplant.cn/tu/13361999
O. chinensis H.S.Lo	Jia-Hua Cai	13403195	https://ppbc.iplant.cn/tu/13403195
O. guizhouensis C.D.Yang & G.Q.Gou	Not available	Not available	Yang CD, He XZ, Gou GQ (2018) *Ophiorrhiza guizhouensis* (Rubiaceae), a new species from Guizhou Province, southwestern China. PhytoKeys 95: 121–126. https://doi.org/10.3897/phytokeys.95.22506

O. grandibracteolata F.C.How ex H.S.Lo	K.M. Feng 13521	00095972	https://kiki.huh.harvard.edu/databases/specimen_search.php?mode=details&id=14254
O. longipes Craib	A.F.G. Kerr 6162	K000740663	https://species.data.kew.org/species/urn:lsid:ipni.org:names:758508-1
O. sp.	s.coll. 1535	E00847692	https://data.rbge.org.uk/search/herbarium/?specimen_num=847183&filename=E00847692.zip&cfg=zoom.cfg
