# Supplementary figures and images for: Ophiorrhiza xishuiensis (Rubiaceae), a new species endemic to Guizhou, Sichuan, and Chongqing, China
Source: PhytoKeys. 2026 May 29;275:163–76. doi: 10.3897/phytokeys.275.189435 (PMC13241921; doi:10.3897/phytokeys.275.189435)

Plants of the new species cultivated in the Guizhou Botanical Garden

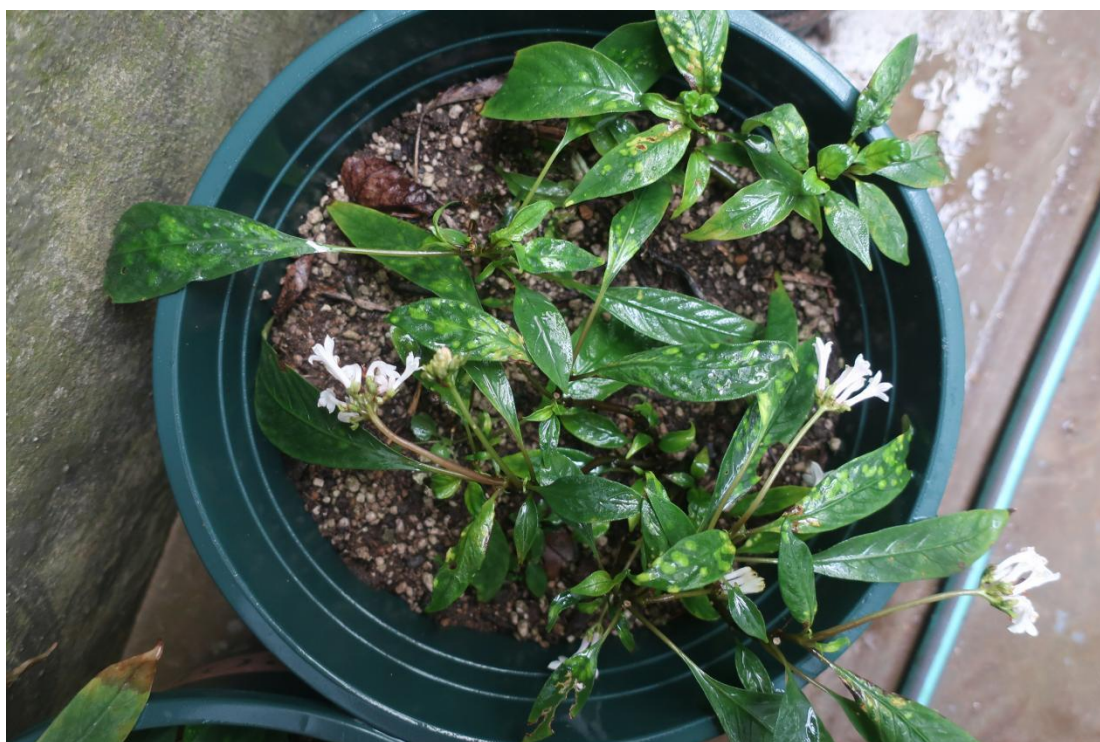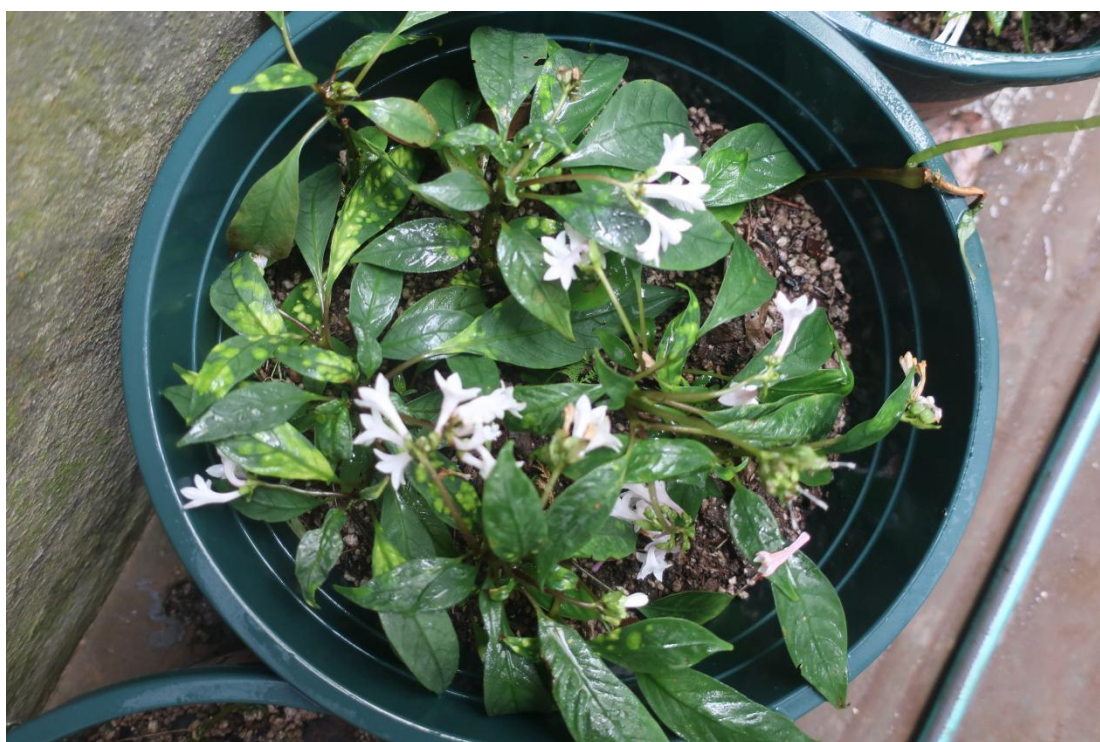

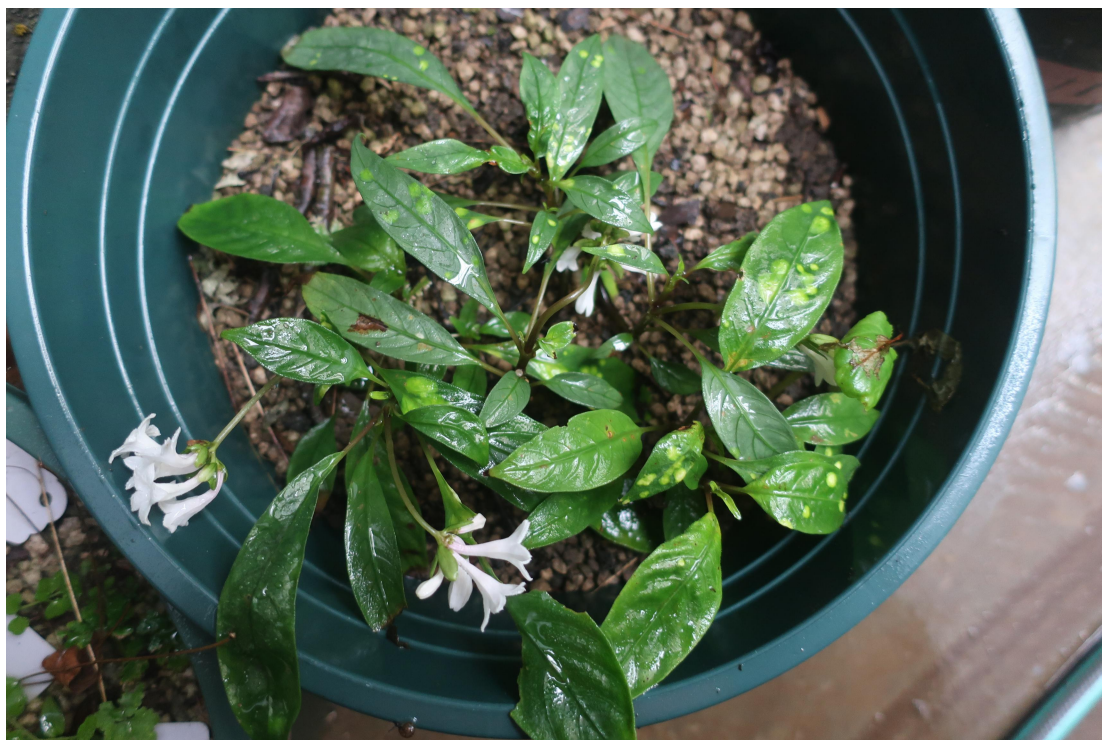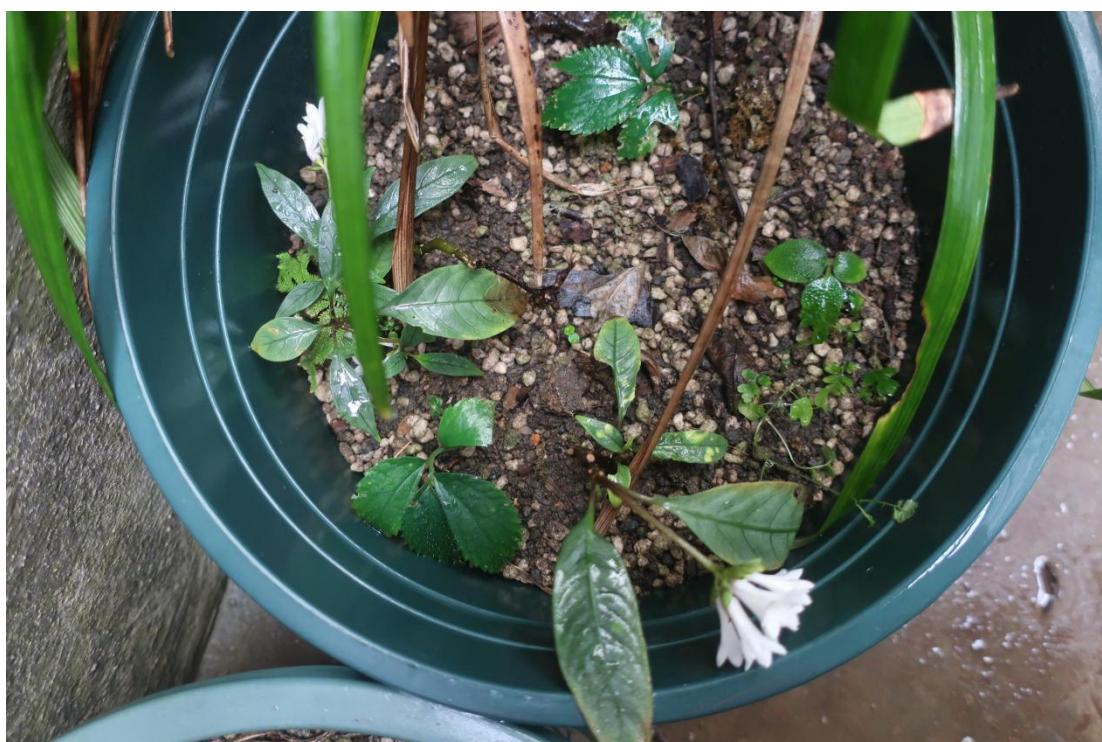

Supplement: Supplementary material 2 — Supplementary figures S1–S4 [file phytokeys-275-163_article-189435__-s002.pdf]
